# Late industrialisation and global value chains under platform capitalism

**DOI:** 10.1007/s40812-022-00240-2

**Published:** 2022-11-28

**Authors:** Wim Naudé

**Affiliations:** 1 TIME Research Area, RWTH Aachen University, Aachen, Germany; 2 University of Johannesburg, Johannesburg, South Africa

**Keywords:** Digitisation, Digital platforms, GVCs, Industrialisation, Competition policy, O25, O33, O14, L52

## Abstract

The digital (or 4th industrial) revolution has made industrialisation harder by being less consequential for structural transformation than was initially hoped. The rise of digital platform capitalism and its relation to global value chains (GVCs) is responsible for this. This paper explains why diminished expectations of the 4th industrial revolution are justified and how this is due to digital platforms as intellectual monopolies that are reconfiguring GVCs—and by this, making industrialisation harder. As such, the paper contributes to the research lacuna on the relationship between GVCs and digital platform capitalism. The implications for late industrialisation are identified, and broad recommendations for industrial policies are made.

## Introduction

The digital “revolution” that emerged out of the technology of World War II and grew in significance in the 1980s, first with the personal computer, then in the 1990s with the World Wide Web, and eventually in the 2000s with mobile connectivity, artificial intelligence (AI) and big data, has significantly boosted and reconfigured international trade. Most importantly, aided by declining transport costs and trade liberalisation, the digital revolution has enabled the fragmentation of international trade,^1^ as reflected in the ubiquity of global value chains (GVCs). A GVC^2^ can be defined as “the series of stages in the production of a product or service for sale to consumer” where “at least two stages are in different countries” (World Bank, 2020: 17). The extent of a country or region’s participation in GVCs is measured by calculating a GVC participation rate.^3^ Since the 1970s, the GVC participation rate for most countries has increased significantly, and participating in and upgrading in GVCs are widely seen as being necessary for industrialisation^4^ (Hauge, 2020; UNCTAD, 2013).

GVCs have, however, more accurately, been a “mixed blessing” for late industrialising countries (Pahl & Timmer, 2020; Rodrik, 2018). For instance, using input–output table data on 58 economies over the period 1970 to 2008, Pahl and Timmer (2020) found that participation in GVCs helped countries to raise their productivity significantly but that there was a “negative association between GVC participation and employment growth” (p. 1685). Moreover, the fragmentation of production and trade into GVCs has reduced the industrial policy space for late industrialising countries (Hauge, 2020).

The rise of digital platforms over the past decade, a manifestation of what has been termed digital platform capitalism, has raised further concerns about the costs of GVCs for late industrialising countries, which may imply that even the positive productivity and growth effects may diminish and that their policy space may further shrink (Bonina et al., 2021; Grabher & van Tuijl, 2020). According to Kenney and Zysman (2016, p. 61), digital platforms are reconfiguring “globalisation itself.” Furthermore, according to Coveri et al. (2021: 3), digital platforms represent a “data-driven evolution of the transnational corporation” that extends their power and influence—and concentration. While digital platforms as technology may, as technological advances did in the past, make participation by late industrialisation countries in world trade easier, it may also further complicate industrial policy and hence the industrialisation and catch-up growth of developing countries.

The evaluation of the relative costs and benefits of digital platform capitalism for late industrialising countries is, however, hampered by insufficient research on the relationship between GVCs and digital platform capitalism. As Lundquist and Kang (2021: 179) recently concluded, “the interaction between the digital economy and GVCs is not well explored.” Loonam and O’Regan (2022: 161) found that “there remains a lack of empirical understanding of how digital platforms can enable GVC strategy.” Because of these lacunas, the development consequences of digital platforms “are not entirely understood” (Koskinen et al., 2019: 3). Particularly, “there is far more work to be done to explore the ‘dark side’ of platforms for development” (Bonina et al., 2021: 893).

By focusing on the relationship between technologies such as digital platforms and GVCs and the consequences for late industrialising countries, the purpose of this paper is to contribute towards addressing this research gap. Specifically, the paper contributes to understanding the development consequences of digital platforms—and the dark side of platforms for late industrialising countries. It argues that given the relationship between GVCs and digital platforms, there are three requirements for relevant industrial policies in late industrialising countries.

The argument is constructed as follows. First (in Sect. 2), a critical description is provided of the digital revolution that has resulted in the technologies allowing the rise of both GVCs and digital platforms and platform capitalism. Then, in Sect. 3 it is argued that despite these technologies, there are diminished expectations of the 4th industrial revolution. In Sect. 4 it is explained how these technologies and their use by digital platforms make late industrialisation, including participation in GVCs, harder. Finally, in Sect. 5, three requirements for relevant digital industrial policies to support industrialisation and appropriate GVC participation in late industrialising countries are presented and discussed. Section 6 concludes.

## Background: the digital revolution

This section describes the digital revolution and its resulting digitalisation and digital transformation^5^ of the world economy.

Before doing so, it is necessary to describe and define what is meant by digital platforms and platform capitalism.

### Key concepts

Platform capitalism refers to the growing dominance of digital platform firms in the global economy.^6^ Digital platform firms are online firms that intermediate transactions between businesses, consumers, and peers and extract rent from this (Parente et al., 2018). Formally, a digital platform is “a delocalised marketplace, the foundation of which is a distinct technological core and a set of self-imposed rules (defining its functionality for its complementors and users) that acquires data from complementors and users while facilitating market transactions” (Butollo, 2019, p. 8). Through their technology and business models, digital platforms link consumers and producers. See Liang et al. (2022) for a critical discussion of definitions of platform capitalism, the platform economy, and related terms.

Digital platform firms have become hugely influential: they disrupt traditional businesses wherever they compete (Ojala et al., 2018). By 2020, more than 10,000 digital platforms were active in Europe (Cabral et al., 2021). Eight of the ten most valuable firms globally, based on market capitalisation, were digital platform firms. In 2019, global digital platform revenue was estimated at US$ 3.8 trillion in value, of which 48% was generated in Asia, 22% in the USA, 12% in the Euro area and 18% in the rest of the world (Lundquist & Kang, 2021). Asia thus leads in the development of the digital platform economy. The COVID-19 pandemic that broke out at the end of 2019 has accelerated the dominance of these firms (Kenney & Zysman, 2020).

Market competition is increasingly taking place *against* digital platforms (e.g., Apple’s watch competing against the Swiss watch industry), *between* digital platforms (e.g., between Apple and Google), or *on* digital platforms^7^ (e.g., between app-developers).

Platform capitalism, as described in the previous paragraphs, is the outcome of the digital revolution,^8^ sometimes also referred to as the 4th Industrial Revolution^9^ (4IR) (Schwab, 2016). While there have been many analyses of industrial policies for late industrialisers considering the digital revolution or 4IR, these have so far stopped short of dealing with the implications of platform capitalism, with the consequences of the changing landscape of competition and GVC participation brought about by digital platforms. The *Oxford Handbook on Industrial Policy* (Oqubay et al., 2020), for instance, have no chapter on industrial policy in an era of big data, and only one chapter concerning digital technologies.

### Connectivity

The digital revolution refers to the acceleration in digitisation and digital transformation of society through technological innovation, including innovation in hardware and software technologies. It has gone through various waves, with the most recent third wave “*a move from a model of accessing the Internet and other networks almost exclusively *via* a desktop computer to alternative forms of distributed information technologies, such as smartphones, wearable computers, and sensors and microprocessors embedded in everyday objects*” (Manwaring & Clarke, 2015, p. 586). The number of sensors increased from an estimated 10 million in 2007 to 15 billion by 2015, while more than 1 trillion semiconductors and integrated circuits were sold by 2018 (Patsavellas & Salonitis, 2019). This third wave has seen exponential increases in computing power, as tracked by Moore’s Law, exponential declines in the cost of computing, and unprecedented connectivity via the distributed information technologies just mentioned (sensors, smartphones), as well as cloud computing^10^ and the “worldwide deployment of more than 400 fibre submarine cables (SMCs) over the period 1990–2018, transmitting more than 99% of international telecommunications” (Cariolle, 2021, p. 2).

The combination of cheap PCs, mobile phones, and sensors, all connected to the Internet with its expanding SMC connections, has led to the Internet of Things with exponential growth in data creation (Patsavellas & Salonitis, 2019). The creation of big data^11^ has increased the complexity of industrialisation in two ways—by enabling cyber-physical systems (CPS) and digital platform firms with business models based on data and AI (Hirsch-Kreinsen, 2016).

### Cyber-physical systems

Cyber-Physical Systems (CPS), which integrate sensors, Internet data, and algorithms with machinery, improve efficiency in manufacturing by enabling continuous monitoring, feedback, and control of production—often independently of human oversight. One of the significant impacts has been to advance the paradigm of lean production (Maffei et al., 2019).

Lean production (lean manufacturing), which, borrowing from Japanese management practices, aims to use technology to increase efficiency in production by reducing stock holdings and waste, speeding up supply chains, predicting maintenance, and providing better value to the consumer (Sundar et al., 2014). Examples include the use of sensors and cloud computing to help drive automation and predictive maintenance, of new connection protocol standards to contribute to better monitoring and use of resources, of mobile connectivity, to facilitate customer interaction and customisation, and of 3D printing to help with rapid prototyping (Patsavellas & Salonitis, 2019).

Lean production is, however, notoriously complex due to its high interdependencies, need for effective coordination and dependence on the error-free operation of digital tools (Butollo, 2019). Thus, while CPS and lean production can improve manufacturing efficiency when firms can get it right (Brynjolfsson & McElheran, 2019), the general roll-out across firms and countries and underpinning of CPS has been slower than was initially expected or hoped.

A further reason for the slow adoption and low impact of CPS and lean production is that, as Hatton and Webb (2020) pointed out, within the Internet-of-Things, there is no universal standard (yet) for connecting distributed devices as there is, for instance, for Wi-Fi.

### Business models based on data and artificial intelligence (AI)

The rise in connectivity and data through cloud computing has facilitated the establishment and scaling up of digital platform firms that use data and AI at the core of their business model—including the governance of the value chains that they coordinate (Rikap, 2022). Global platform giants, such as Google, Amazon, and Facebook, amongst others, are the largest investors in developing and deploying AI. To them, AI is a handy tool to extract intelligence from the mass of big data and use this to create intellectual monopolies—see the discussion in Rikap (2022).

Although the term AI was coined in 1956 (Moor, 2006), it was only in the last 12 years or so that modern AI, based on Machine Learning (ML), came into use, following the mentioned availability of big data, as well as advances in computing, such as the development of efficient Graphical Processing Units (GPUs) for computers in the video gaming industry, and the elaboration of algorithmic techniques that could combine these to allow computer programs to recognise patterns in data and make predictions, and learn to improve these over time (Cano, 2017; Hinton & Salakhutdinov, 2006; Hinton et al., 2006; LeCun et al., 2015).

With its ability to spot patterns and make predictions, AI is used mainly by large platform firms with access to big data and computing and IT skills resources in applications such as recommender systems, targeted advertising, chatbots, search engines, and translation. The development and use of AI are heavily concentrated in the USA and China, where the world’s leading digital platforms are located and where the vast bulk of AI patents are held (WIPO, 2019).

AI is yet to have a significant impact on manufacturing and the economies of developing countries. There have, however, been expectations that AI will revolutionise manufacturing, for instance, through predicting when maintenance is needed (which is already possible) to enable autonomous driving and self-organising factories (not yet implementable at scale and cost) (MIT, 2020). AI is, however, not diffusing very fast, nor is its technical potential yet attained. AI remains expensive, primarily available only to large digital platforms and out of reach of small businesses, which do not have access to large enough datasets to train AI models, as well as not safe or ethical enough, and increasingly burdened in its implementation by (expensive) regulations (Bergstein, 2019; Farboodi et al., 2019; Naudé, 2021). Lee and Lee (2021), using USPTO patent data, found that most 4IR technologies, and AI specifically, have had less of a broad impact on subsequent innovations than earlier 3IR technologies had, concluding that AI “may not be counted as a new general-purpose technology” (Lee & Lee, 2021, p. 137).

Furthermore, in addition to these unfavourable cost–benefit features of AI, the technology has come under scrutiny, not only for posing possible long-term existential threats (Bostrom, 2014) but having shorter-term negative consequences, such as intrusive surveillance and erosion of privacy, creation of AI weapons, job losses due to automation, higher inequality, and fuelling discrimination and biased policy-making (Feldstein 2019; Frey & Osborne, 2017; Korinek & Stiglitz, 2017; Russel et al., 2015).

These downsides of AI raise the risk to firms from using AI and hence increase the complexity of industrialisation and of crafting industrial policies in the digital age. It moreover suggests that much more investment, development and regulations may be needed in AI and its supportive infrastructure before it can be scaled through the manufacturing sector and benefit producers in developing countries. Just as CPS and AI-driven business models of digital platforms make industrialisation harder, a much older technology—3D printing—has been similarly hyped as a 4IR technology but has been found to raise complexity and have less than hoped-for impacts. 3D printing (or additive manufacturing) has been hyped as a “spark” of the new (4IR) industrial revolution (Weller et al., 2015, p. 43), even though it is essentially a 1980s technology.

In recent years, it has improved by being linked to the digital economy through the separation of product design from manufacturing capabilities and use together with 3D CAD software and Computer Numerical Control^12^ (CNC) machining (Berman, 2012; HUBS, 2020). The potential of 3D printing as a spark of the new industrial revolution is discussed in Karlgraad (2011), Anderson (2012) and Rayna et al. (2015), amongst others.

For late industrialising countries, the attractiveness of 3D printing is in the potentially to avoid realising economies of scale in production, obtaining cheaper inputs and spare parts in manufacturing, being able to make more affordable and faster prototypes, reducing assembly costs, and customise production to local demand (Berman, 2012; Khajavi et al., 2014; Kleer & Piller, 2019; Weller et al., 2015).

It is, however, the case that, as with artificial intelligence (AI) that 3D printing is still not widely adopted, mainly due to the high cost of printers, the limited range of materials and colours available, high-energy needs,^13^ the generally insufficient quality of products, and increased knowledge requirements—and copyright implications—posed by the software (Chan et al., 2018; Rayna et al., 2015).

The discussion in the preceding paragraphs suggests that while there has been a digital revolution marked by remarkable advances in various ICT technologies, especially in connecting and combining technologies and their users, the implications for industrialisation have been to make it more complex, harder, and uneven.

## Diminished expectations

In the introduction, the link between GVCs and digital platforms was made, and it was pointed out that the rise of the latter has raised concerns about the costs of GVCs for late industrialising countries. While digital platforms as technology may, as technological advances did in the past, make participation by late industrialisation countries in world trade easier, it may also further complicate industrial policy—and hence the industrialisation and catch-up growth of developing countries.

As the previous section made clear, the digital revolution has raised expectations that it would facilitate industrialisation. In this section, the link to the arguments stated in the introduction is made by discussing why and how these expectations have been severely diminished by digital platform capitalism. There are several reasons why this is the case, which range from differences in the digitalisation intensity of sectors across time and space to disappointments with the monopolistic and declining business dynamic outcomes associated with digital platform capitalism.

Regarding differences in digitalisation, Calvino et al. (2018) proposed a taxonomy to measure the extent to which sectors are being digitised. Their key indicators of digitalisation are (i) ICT investment, (ii) purchases of ICT intermediates, (iii) ICT specialists, and (iv) online sales. Applying this to various sectors, they distinguish between digital and less digital-intensive industries. Their overall (global) results (Calvino et al., 2018, p. 31) distinguish between low, medium–low, medium–high, and high digital-intensive sectors and compare this between 2001–2003 and 2013–2015.

They found that unlike manufacturing, where most sectors are either medium–low or medium–high digital intensity, it is the services sectors that have been “going digital”—particularly telecommunications, IT and other information services, finance and insurance, legal and accounting activities, scientific research, advertising and market research and administrative and support services are all highly digitally intensive sectors. Thus, compared to services, manufacturing has not seen similar digitalisation—the relative digital intensity of computer and optical products even declined from high in the 2001–2003 period to medium–high in 2013–2015.

Why may this be the case? One reason may be that “*since a disruption of established processes is laborious and risky, the implementation of new digital technologies has tended to be pursued rather in an incremental manner than as radical change*” (Butollo, 2019, p. 3).

Other related reasons could be the increased complexity of utilising the new technologies and the risks that the gains from these technologies may not be significant or sustainable. This raises a relevant consideration for late industrialising countries—namely that the notion that the digital revolution will have sweeping changes to manufacturing (industrie 4.0) and will be a force for structural change may have been exaggerated, and that diminished expectations may be in order. According to Deichmann et al., (2016, pp. 21–22), “*Globally, productivity growth has slowed, inequality is a rising concern not just in rich but also in low- and middle-income countries, and technology has not led to the widespread improvements in governance that many had predicted*.*”*

This quote from Deichmann et al. (2016) alludes to several reasons for justifying diminished expectations of the 4IR. There are at least four such reasons. Three of these are generally well recognised and relate to the lack of aggregate improvements in development outcomes (growth, jobs, productivity) and (data) governance problems, as noted in the Deichmann et al. quote, and two which are, per the arguments in the introduction, somewhat neglected—relating to the rise of digital platform firms. The remainder of this sub-section will elaborate on these reasons, and the next section, Sect. 4, will elaborate on the implications of digital platforms.

The first reason for justifying diminished expectations of the 4IR is that industrial policies will have to contend with the likelihood that digital technologies and their business model consequences will have less of an impact on productivity growth than earlier technologies had during the 1st and 2nd industrial revolutions. It is a well-established fact that productivity growth in advanced economies has slowed since the 1970s, particularly in manufacturing, since around 2007–2010, when the development of the digital economy and digital platforms accelerated (Lawrence, 2017). This likely reflects that “ongoing innovation has been less potent in boosting productivity growth compared to earlier decades of the post-war era” (Gordon & Sayed, 2020, p. 50). See Bloom et al. (2017), who document the decline in USA research productivity.

A second reason for justifying diminished expectations of the 4IR is that digital industrialisation will not automatically lead to sustainable or green industrialisation. Digitalisation poses many new challenges for achieving the Sustainable Development Goals (SDGs) relating to climate change actions. There are concerns about the high carbon footprint of many new digital technologies, including cloud computing, artificial intelligence (AI) and distributed ledger technologies (DLT) such as bitcoin.

For example, cloud computing has led to a proliferation of data management centres which will, according to estimates, consume between 13 and 51% of global electricity in 2030 and will be responsible by then for 23% of all CO_2_ (Patsavellas & Salonitis, 2019). The energy needs of 3D printing are “100 times higher than that of traditional manufacturing” (Chan et al., 2018, p. 156) and training a large AI model emits as much CO_2_ as five large American motor vehicles over their entire lifetimes (Naudé, 2021).

The third reason for justifying diminished expectations of the 4IR is that manufacturing development can contribute to growing inequalities and that manufacturing has become less of a job creator than in the past. Even without factoring in the rise of digital platforms, the digitisation of manufacturing has raised the possibility that automation could lead to the displacement of low and medium-skilled jobs and complementing of high-skilled jobs (Balsmeier & Woerter, 2019), meaning that the digitisation of manufacturing could create relatively more jobs and wage increases in high-income, high-skilled industrialised countries, and lead to job losses in developing countries—exacerbating global inequalities.

With the rise of digital platforms, the added complication is that platforms accelerate the automation of jobs and promote potentially poorer-quality jobs. For example, by “taskification”, jobs are turned into short-term tasks or gigs where labour sells its expertise on various online gig platforms—effectively establishing global value chains in labour markets (Grabher & van Tuijl, 2020). Both automation and the taskification of jobs thus make it more difficult for latecomer industrialisers to realise the traditional employment benefits from manufacturing growth.

The fourth reason for justifying diminished expectations of the 4IR is that whereas in earlier industrial revolutions, new business formation played a role in introducing and commercialising new technologies and, in effect driving structural transformation (Gries & Naudé, 2010), the “burden of knowledge” of participating in the digital economy and leveraging the network economies inherent in multi-sided platforms and their underlying hardware, may reduce new start-ups and new venture creation in the high-tech manufacturing industry—in other words, the burden of knowledge effect can reduce the role of new ventures to push structural transformation through creative destruction (Astebro et al., 2020).

In conclusion, the technology of the digital revolution complicates the process of industrialisation, as it poses high requirements for complementary skills, capabilities, and infrastructure and tends to be less consequential for structural transformation than was initially hoped. Diminished expectations of the 4IR imply that late industrialising countries not only need to overcome the complexities of technology but find ways of better leveraging these for better impact and deal with the potential adverse consequences of uneven digital capabilities between countries. The latter requires that late industrialising countries understand and respond to the rise of digital platform firms. How digital platform firms make industrialisation, primarily through participation in GVCs, harder is explained in the next section.

## Industrialisation is becoming harder

The digital revolution discussed in the previous section has increased the complexity of development along three interrelated dimensions that will be discussed in the sub-sections that follow.

### Software is eating the world

The first way platform capitalism complicates industrialisation is that digital platforms have shown themselves deadly competitors, when they face off against traditional pipeline brick-and-mortar businesses. Examples are Amazon and Netflix out-competing Borders and Blockbuster and driving them out of the market.

The history of competition of digital platforms against traditional “pipeline” businesses caused Parker et al. (2016) to conclude that “When a platform enters a pipeline firm’s market, the platform almost always wins.” Given that the most valuable assets of digital platforms are the intangible data and algorithms on which their business models are built, the oft superiority of digital platforms against pipeline producers inspired Andreessen (2011) to coin the phrase “software is eating the world.”

With network effects leading to the capture and domination of markets by one or a few incumbent firms, there is a significant advantage to being a first-mover: first movers, therefore, tend to capture markets and may be challenging to compete against later established firms. The literature on the economics of digital platforms as leveraging transactions on multiple sides has emphasised their strategies to increase the number of users on the platform (Rysman, 2009). Rochet and Tirole (2003) explain how a platform can use differential pricing to subsidise users on the one side of the market, to make the platform more attractive to users on the other side (for example, charging merchants and not consumers a fee for the use of credit cards to increase the number of users of credit cards, making it more attractive for merchants to accept credit cards). With data enabling them to provide better quality services and products, digital platforms aim their technology and business model innovations more effectively away from the needs of businesses and governments towards the need of the consumer, a trend that has been labelled the “consumerisation of technologies” (Sundararajan, 2014, p. 4).

In this type of competition and consumer-oriented innovation, digital platforms often enjoy an advantage in being supported by *patient* finance, that is, the commitment of substantial financial resources even as these platforms may not be making any profits for an extended period. Patient finance has the objective of establishing long-term dominance in a market. Thus, given the presence of network effects and first-mover advantages, it is willing to absorb losses during the expansionary phase (Foster & Azmeh, 2020). Amazon, for instance, established in 1994, made its first annual profit in 2003. Patient finance has been essential in supporting the rise of digital platforms in the USA and China. In the latter, it had been both western Venture Capital (VC) funds as well as state finance that supported the rise of Chinese digital platforms, such as Baidu, Alibaba and Tencent (the BATs) (Foster & Azmeh, 2020).

In addition to efficiency, first-mover, and network effects from harnessing big data, and the support of patient finance, digital platforms are difficult to compete against for traditional firms. One reason is that they often engage in “shapeshifting”, which refers to platforms moving into markets non-related to their original core business, often even entering domains traditionally that of the state (Rikap, 2022; Teng & Jacobides, 2021). This makes anticipation and regulation of their entry difficult. Examples are Apple competing against the watch industry with its Apple Watch, Google venturing into the market for autonomous vehicles, and Amazon into space exploration.

A second reason why traditional firms find competition against digital platforms difficult, and perhaps the most fundamental, is due to the intellectual monopoly capitalism on which they are based (Pagano, 2014). This consist of “knowledge predation” in their platform ecosystem and hoarding of intellectual property rights (IPR) (Rikap & Lundvall, 2020). An example is Amazon which by 2018 had 10,000 patents, which only shared 0.13% even though it is involved in various innovation networks and had co-authored scientific papers with more than 750 organisations (Rikap & Lundvall, 2020: 3).

As a result of the competitive dangers posed by digital platform firms, they spur traditional non-platform firms to significantly adapt their corporate strategies to compete more effectively- raising the complexity of doing business for developing country firms.

Traditional firms typically react in three ways. One, they will implement significant cost-saving measures; two, they will try to make their business model more flexible and customer-oriented—adopting features of platforms; and three, they may start joint businesses with digital platform firms (Parente et al., 2018). An example in the latter regard is the 2019 announced strategic partnership between Volkswagen and Amazon Web Services (AWS), which follows Volkswagen’s partnerships with Microsoft Azure, and which created the Volkswagen Automotive Cloud (Butollo, 2019).

In these reactions, engagement in GVCs becomes more complex due to the changes it requires in business models. Grabher and van Tuijl (2020, pp. 1009–1010) discuss how digital platforms upends the business model of GVC participation. These are that platforms tend to de-emphasise physical production in favour of users and matches; that a platform has more nuanced control imperatives compared to the more hierarchical governance in GVCs; and that firms in platforms are more concerned with leveraging resources outside the firm than internal to the firm—i.e., “invert” the firm (Parker et al., 2016). This complicates industrialisation by essentially requiring new models for doing business and creating value. Implementing such new business models often faces intractable problems in organisational inertia, sunk investment costs, and vested interests.

Furthermore, these typical reactions of traditional firms against the competitive dangers of digital platform firms often fail to consider that digital platform capitalism is characterised by digital platform firms achieving not only market power through the strategies mentioned above but also significant non-market power—through extending control over governments, labour, suppliers, and others (Coveri et al., 2021).

### Goliath *and* Goliath

Irrespective of the type of digital platform, their business models are set up around digital artefacts or artefacts. A digital artefact is “*a product or service either embodied in information and communication technologies or enabled by them*” (Briel et al., 2018, p. 278). Digital artefacts are delivered through a modular architecture and, as mentioned, the utilisation of large volumes of data, i.e., other digital artefacts.

The modular architecture of digital platforms consists typically of four layers, namely a content layer, a service layer, a network layer, and a device layer (Yoo et al., 2010). Ojala et al. (2018) use Netflix as an example to illustrate this modular architecture, where the content layer is provided by film studios, the service layer is the streaming of content provided by Netflix, the network layer by the providers of internet access, and the device layer provided by the hardware devices required on which to watch Netflix.

This example illustrates that digital platform firms need to coordinate the activities of many potentially unrelated service providers across their platform—in effect, it needs to achieve synergies between various prominent role players, a “Goliath and Goliath” cooperation and coordination. This requirement presents a complex challenge even in the most advanced economies (Ojala et al., 2018).

Even if a Goliath and Goliath cooperation can be coordinated by a digital platform, it would still require a Goliath digital institution to underpin this cooperation. Digital institutions that underpin platform capitalism are evolving but already include reputation systems, digital rights management technologies, digital identity verification, reputation and credit scoring systems, distributed ledger technologies, cryptocurrencies, and non-fungible tokens—amongst others.

These digital institutions, most often than not, are “de facto, subsuming government-mediated intellectual property laws” (Sundararajan, 2014, p. 4) and even, to an extent “, putting the nation-state model under serious strain in all sorts of ways” (Bartlett, 2017, p. 297). The Goliath and Goliath cooperation and underpinning required for the rise of digital platforms are therefore considerable and still outside the scope and perhaps even the desire of most late industrialising countries.

### Goliath *vs* Goliath

The third way platform capitalism complicates industrialisation is that, even if late industrialising countries establish home-grown digital platforms, adopt features of digital platforms, or build appropriate digital institutions, they will have to engage in platform-to-platform competition. Here, they are far behind in experience and lessons learned. In advanced economies and China, digital platforms compete against one another. The competition of the large global digital platforms has been described as “Goliath vs Goliath.” An example is Amazon and Google competing for advertising revenue or Apple taking legal action against alleged intellectual property appropriation by Google (Foroohar, 2019).

Often large incumbent digital platforms will compete against newly established, growing digital platforms that try to unsettle them from their dominant position. One strategy is to try and provide a better service to attract the users of the incumbent platform to switch. Eisenmann et al. (2011) discuss the example of Sony’s PlayStation, which out-competed Nintendo’s Super Nintendo Entertainment System (SNES) by offering users a 32-bit processor and 3D graphics, which was better than SNES 2D-graphics and 16-bit processor. The new entrant platform needs access to significant financial and human resources to provide a superior product or service. For developing countries, this, as well as the requirement to coordinate and put in place a modular architecture dependent on the inputs of many other firms, present significant obstacles.

Another strategy one platform will use against another is “platform envelopment.” This is “*entry by one platform provider into another’s market by bundling its own platform’s functionality with that of the targets to leverage shared user relationships and common components*” (Eisenmann et al., 2011, p. 1271). An example is Google “*which has entered many platform markets by linking new products to its search platform, including online payment services (Google Checkout), productivity software (Google Docs), Web browser software (Chrome), and mobile phone operating systems (Android)*” (Eisenmann et al., 2011, p. 1271).

### Five-star bombs and other dirty tricks

The fourth way platform capitalism complicates industrialisation is the competition digital platforms create between third-party entrepreneurs, such as app developers or online retailers, using its digital infrastructure. Examples are app developers on the Apple Store, retailers on Amazon Market Place and Facebook’s Marketplace.

The platforms set the terms of this competition, including, importantly for sellers, how high up in online search rankings their product or service will appear. This has had many complications, most often to the detriment of small firms,^14^ or freelancers operating on the platform. These complications have arisen from the platform both making the rules and competing with other users, thus not being a neutral arbiter, and from the manipulation and misuse of the rules by users against each other.

Dzieza (2018) describes the troubles that small businesses and freelancers may encounter by competing on a large digital platform such as Amazon. These have even been given labels such as “the five-star bomb,” “hijacking,” “defacement,” and “phoney fire.” A “five-star bomb”, for instance, is when “a seller pays someone to write obviously fraudulent five-star reviews for a competitor’s listings and hopes Amazon cracks down” (Dzieza, 2018).

If Amazon cracks down, an entrepreneur may suddenly find their account suspended and their business unable to operate. Small firms do not have effective recourse if, in the case of a five-star bomb or another dirty trick, Amazon unfairly shuts its business down, as they agree to an arbitration procedure when signing up to the platform. This arbitration procedure, however is biased in favour of Amazon "by discouraging sellers who lack the money, time and energy to take on the company” (Soper, 2021).

Moreover, Amazon may be inclined to crack down more fiercely on 3rd party sellers on its platform if those sellers offer products that compete with one of Amazon's own (Weise, 2019). Moreover, there are cases reported where Amazon copied the products of successful 3rd party sellers and sold them at a discount on the platform to steal its business or avoid competition (Addady, 2016).

There is nothing inherently wrong with a competitive online marketplace, and many freelancers and entrepreneurs run successful businesses on such platforms. Increasingly, given the spread of digital platforms to developing countries and the development of more home-grown digital platforms in these countries, more and more businesses, including new ventures supporting industrialisation, will be doing business on a digital platform. Their ultimate lack of control over their business and the inequities of platform competition, however, implies a significant degree of dependence on foreign platform owners, which may not be the best approach to sustainable industrialisation.

### Kill zones and GCV substitution

The fifth way platform capitalism complicates industrialisation is that local business dynamism tends to taper off in the presence of large digital platforms. Four mechanisms at work here are one, the lack of competitiveness of local firms against the more effective and customer-oriented platform model with its network economies. Two, the start-up of new firms, the engine of innovation-driven growth, declines in the presence of large digital platforms. Three, that existing small and medium enterprises (SMEs) in late industrialising countries may lack the capabilities to partake effectively in the digital platform economy; and fourth, that.

The lack of competitiveness of traditional local firms against platforms has been discussed in Sect. 4.1. In the case of late industrialising countries should digital platforms enter more into these markets and become more prominent in future, they may out-compete local small businesses, as they have done in advanced economies. This will be not only due to data network effects but also because platforms may be able to fill gaps in developing economies and even do so better and more cheaply than traditional local businesses. Platforms, particularly peer-to-peer (P2P) platforms, may be able to overcome cultural barriers through the internet, establishing trust through rating and feedback systems, and bringing down the cost of matching buyers and sellers significantly (Parente et al., 2018):

Local business dynamism also tends to taper off when large digital platforms firms create “kill zones.” This happens when new ventures are likely to be taken over by digital platforms or their intellectual property appropriated if they pose a threat (Foroohar, 2019; Kamepalli et al., 2021). Global platform giants have been engaging in an active spree of M&A—the number of firms taken over by USA-based digital platform giants runs into the hundreds.

Likewise, Chinese digital platforms have taken over many firms in China and the East Asian region, an example being Alibaba taking over Indian-based firm PayTm (Foster & Azmeh, 2020). While part of the reason for buying up new start-ups is to gobble up potential competitor firms (e.g., Facebook’s acquisitions of Snapchat, Instagram, and WhatsApp), it is also done to obtain access to more and diverse data, which allows for recombination and aggregation of data from which new value can be extracted (Li et al., 2019).

Many start-ups, aware of these kill zones, accordingly, never plan to establish a long-run, sustainable enterprise but merely aim to enter the market with a product or service that will attract the attention of a digital platform so that they can be bought up even before launching an IPO, such as Instagram (Kamepalli et al., 2021).

A third mechanism through which the rise of digital platforms may reduce local business dynamism in late-industrialising countries is that the aspects of their business model may make it difficult, given the capabilities of most developing countries’ SMEs, to take part in the platform economy. Lundquist and Kang (2021) discuss the challenges in this regard. These include that SMEs often lack sufficient training and skills in ICT and digital technologies to harness the benefits of digital platforms, that digital platform participation may be subject to high fixed costs which many SMEs cannot afford, that the algorithms and rating systems used by digital platforms may be biased against SMEs; digital platform consolidation (when one or very few platforms come to dominate a market as a result of network effects) may reduce the advantages for SMEs as the latter do not have any bargaining power in the form of alternatives. These challenges may hinder SMEs from participating in GVCs.

The impacts of digital platforms on local business dynamism may also have an adverse long-run effect on developing countries’ participation in GVCs. This is because the competition from digital platforms may truncate or reduce the number of large firms that survive or emerge in a developing country. This is important as a stylised fact of exporting is that the bulk of any country’s exports tends to be done by only a few large firms—export superstars (Freund & Pierola, 2015). Individual firms are therefore crucial for exporting, and would developing countries lose their significant export superstars due to digital platform capitalism it will negatively affect their ability to export.

Finally, as Lundquist and Kang (2021: 193) also discuss, “digital platforms may be substituting for traditional GVCs” with the adverse consequence that participants—developing country SMEs—do not benefit as much as they did under traditional GVCs. One of the benefits of traditional GVCs that often gets lost on digital platform intermediate trade is the transfer of knowledge and know-how, hence the opportunity to learn, as the party governing the digital platform is often reluctant to share information and technology.

The implication of the above is, as concluded by the World Bank (2020: 141), that digital platforms “make it easier, then, to connect, but harder to compete.”

### Gatekeepers

The sixth way platform capitalism complicates industrialisation is that digital platform firms have become par excellence the lobbying firms of the present generation. Google and Amazon’s close relationship with the US Department of Defence has been noted (Sadowski, 2020). China’s BAT’s (Baidu, Alibaba and Tencent), although not government-owned, have a close relationship with the Chinese communist party. Given the centrality of data and new technologies based on data to the business models of large digital platforms, they have a strong interest in weak data and intellectual property protection and engage in extensive and well-coordinated lobbying and legal efforts to influence policymaking (Foroohar, 2019).

One of the practices that digital platform firms want to protect is that of *digital enclosure*. Digital enclosure refers to the creative but predatory use of software licenses to obtain control and access users’ data. For example, when a factory owner purchases a smart machine, they would typically obtain ownership over the physical object, not the embedded software, which through the licensing agreement, is leased or rented. This allows the owner of the software access to the use of the machine—even to the extent of shutting it down (Sadowski, 2020).

The influence that digital platforms have over the competitive landscape and the increasing efforts they exert to gain influence over policymakers is of even more significant concern considering that the largest and most predominant of the digital platforms have in essence become gatekeeping intermediaries^15^ between consumers and producers (and consumers and consumers) (Foroohar, 2019; Cabral et al., 2021). This has made their potential abuse of their position an even graver cause of concern.

### Surveillance capitalism

Finally, platform capitalism complicates industrialisation as there are many new downsides to an economy where data is becoming increasingly valuable, and platforms compete for user attention and data. Digital platforms’ huge hunger for data and the high value that data holds for them has given rise to dubious business models—such as violations of data privacy and data harvesting (gathering data without the knowledge and permission of users) and models that foster digital addiction.

Data harvesting and digital addiction are often two sides of the same coin within platforms’ business models. For instance, if Google or Facebook’s business model depends on selling advertising space (auctioning of space) and data for advertisers, then it has the interest in collecting as much data as possible but also in keeping users engaged on their platform for as long as possible. This result in a digital architecture that plays in on humans’ dopamine centres, causing addiction, resulting in clickbait and false news in a battle to gain as much as possible of users’ attention in a model—where the user of these platforms becomes, in effect, the product. Thus, given that “*showing consumers arousing and sensationalist things [...] will make them stay longer than something truthful and useful [...] If you let a machine learning algorithm loose in these platforms, it will discover that people click more on this kind of content, and therefore the platform will deliver more of it*” (Morton, 2021, p. 143).

With the widespread prevalence of data harvesting and digital addiction, digital platform capitalism has been described as surveillance capitalism—and has been accompanied by the emergence of a surveillance state (Murakami Wood & Monahan, 2019; Srnicek, 2017a, b; Zuboff, 2015). Surveillance capitalism includes not only direct surveillance activities by platforms but “*the manifold and often insidious ways that digital platforms fundamentally transform social practices and relations [...] and setting the terms upon which individuals, organisations, and governments interact*” (Murakami Wood & Monahan, 2019, p. 1). Pasquale (2016) contrasts two narratives of digital capitalism, noting that because the technical press is dependent on advertising revenue from “big tech” they tend to provide an over-optimistic narrative of digital capitalism—including hyping the 4IR.

Considering this, industrialisation in late-industrialising countries faces a novel set of challenges relating to the value, use and misuse of data. Very few African countries have endorsed the African Union’s Convention on Cyber Security and Personal Data Protection at the time of writing. Furthermore, more and more are resorting to utilising new digital technologies to spy on their citizens and restrict access to the Internet. This situation creates uncertainty, distrust, and vulnerabilities, limiting the absorption and use of new digital technologies for domestic industrialisation.

## Implications for industrial policy and global value chain participation

Industrial policy refers to “*any type of selective government intervention or policy that attempts to alter the structure of production in favour of sectors that are expected to offer better prospects for economic growth that would not occur in the absence of such intervention*” (Pack & Saggi, 2006, pp. 1–2). Digital industrial policy refers to industrial policy where the emphasis is on “*new approaches that are relevant to digital technologies and the new business models that are common in the digital economy*” (Foster & Azmeh, 2020, p. 1248). Digital industrial policy should thus not only be concerned with the nature and implications of new digital technologies but also with the business models that they give rise to and how they affect the participation of countries in GVCs and the value that they get from GVCs. More attention is needed in the literature on late industrialisation on this latter aspect and the relationship between GVCs and digital platforms.

Late industrialising countries require very deliberate digital industrial policies to deal with business models such as the digital platform firm, which underpin digital platform capitalism. Such policies need to deal with at least three issues that will subsequently be discussed.

### Avoid marginalisation or capture

As a first requirement to bring digital platforms within the purview of digital industrial policy, developing countries should formulate appropriate responses to the industrial policy responses of leading manufacturing countries, e.g., the USA, Germany, and China. These countries have all, in recent years, in response to the digital revolution, developed new industrial strategies. These will either close policy space and opportunities in late industrialising countries or create more opportunities—or a mix of both.

In the West, the USA is developing smart manufacturing to re-shore jobs outsourced to China and other Asian countries during the latter’s rise. With its Industrie 4.0 strategy, Germany aims to digitise its manufacturing sector, including promoting new business models for manufacturing that would likewise re-shore jobs and shorten value chains.

Germany, and the EU more generally, has also started to pay much more attention to the regulation of USA-based digital platforms and their potential negative consequences for European industries,^16^ for instance, through initiatives such as the General Data Protection Regulation (GDPR), more aggressively bringing anti-competitive actions against the large USA digital platforms, and formulating its own Digital Strategy (Europe’s Digital Decade).

In China, the state uses existing large digital platforms—the BATs—deliberately as an industrial policy tool to support less innovative regional firms to modernise, such as in transport, small-scale manufacturing and regional retail (Foster & Azmeh, 2020). An example of this is the creation of Taobao-villages, which is a consumer-to-consumer (C2C) digital platform owned by Alibaba that sells products made locally in regional areas by small-scale producers (Butollo, 2019).

China’s digital industrialisation ambitions may be consequential for late industrialising countries, such as in Sub-Saharan Africa, as the region is explicitly targeted. China’s vision for global industrial leadership is based on a comprehensive digitisation strategy to attain digital sovereignty and dominance. The establishment and promotion of digital platform giants and their spread into emerging markets like Africa is a central plank of this strategy. The digital giants will cement their leading positions not only through the network economies from the large domestic Chinese market but through locking in the economies of emerging economies into their technology hardware, standards, and cyber governance systems.

China’s industrial policies follow its *Made in China 2025* strategy, which aims to position China as the world's leading high-tech manufacturing hub. Made in China 2025 (MiC2025) has several components with implications for African industrialisation, such as the Belt and Road Initiative (BRI) with its digital counterparts, the Digital Silk Road (DSR), the Internet Plus initiative, and the China Standards 2035 plan (Dekker et al., 2020). As a result of these, China’s most prominent digital platforms are expanding into Africa. Anwar (2017) discusses the case of Alibaba, one of China’s most prominent digital platforms, and how it expands into foreign markets—including those in Sub-Sahara Africa.

Since 2017 Alibaba has expanded its global reach into Sub-Sahara Africa, aiming to create a “pan-African eco-system based on the Alibaba model” and several interlinked initiatives to gain rapid market share across the continent. These include the rolling out of the Electronic World Trade Platform (eWTP) to link African consumers and firms to those in China (it is part of the Digital Silk Road), the Africa Netpreneur Prize (ANPI) to identify promising new businesses for Alibaba to invest in, and the cultivation of close ties to African political leaders (Velluet, 2020). The Alipay mobile payment platform has entered into collaboration agreements with all of Africa’s main payment infrastructures and services, including M-Pesa, Vodacom, Ecobank’s RapidTransfer, Flutterwave and Vodacom (Velluet, 2020).

Whereas Western digital industrial strategies are likely to leave African countries more excluded or marginalised through the withdrawal of manufacturing activity through re-shoring (and reducing their participation in GVCs) and automation in the West, and through the restrictions imposed by the GDPR and other privacy-oriented legislative responses, China’s industrial strategy aims to dominate African economies by locking their economies into China’s technology hardware, standards, and cyber governance systems—and ultimately Chinese-lead GVCs.

### Appropriately regulate digital platforms

A second requirement for digitally relevant industrial policies in late industrialising countries is to regulate global digital platforms so that developing countries can benefit from their presence but avoid their many dangers. Global digital platforms pose risks, but they also have many potential benefits, as illustrated during the COVID-19 pandemic (Kenney & Zysman, 2020).

The various ways digital platforms complicate industrialisation, as set out in Sect. 3 of this paper, suggest that how they compete and their first-mover advantages and dominance of markets may pose the most severe obstacles or dangers for late industrialising countries. The monopoly power that these digital platforms obtain because of data network effects, on the one hand, generates benefits in the form of more efficient products and services for consumers and users of their platform, but on the other, brings with it possible misuses and abuses of this power—such as stifling competition and engaging privacy-eroding surveillance practices.

The challenge for regulators in the age of platform capitalism is how to preserve dynamic competition, i.e., prevent the misuse and abuse of digital platform monopoly power and gatekeeper function (Cabral et al., 2021). Misuses, abuses, and the resulting harms to social welfare may create a very uneven and unfair playing field for late industrialising countries. The EU’s grappling with this challenge indicates the complexity of the matter.

Like late industrialising countries in the developing world, the EU is marginalised as far as global digital platforms are concerned: Europe has no comparable digital platforms to compete with those of the USA and China. As a result, Europe’s digital industrial strategy is to regulate (US and Chinese) platforms. The EU has, for instance, in recent years, in addition to the GDPR, which concerns the use of data, adopted its EU Platform-to-Business (P2B) Regulation (2019), and proposed a Digital Markets Act (DMA) and Digital Services Act (DSA) in December 2020 (Cabral et al., 2021). And it has brought an increasing number of punitive legal cases against global digital platforms—for example, between 2017 and 2019 the EU imposed almost €9 billion in antitrust fines on Google. Despite this, Pianta et al., (2020: 781) conclude that “Europe is showing a continuing inability to confront the monopolistic power at the global level of large US digital firms—Amazon, Apple, Facebook, Google, and Microsoft -in fields such as technological and platform development, 5G technologies, and control over data.”

For late industrialising countries, the EU's regulatory approaches and antitrust fines against global digital platforms, and China’s efforts to impose its standards and governance systems on the worldwide economy, are a signal that digital industrial policy in the age of platforms capitalism will ultimately be concerned with regulations and standards (Li et al., 2019). Herein, regulations and standards about data will be paramount, as data is the oil that fuels the business models of digital platforms. Regulations and standards about data will have to deal with its ownership, sharing, exchange, and privacy protection issues.

The governance of data poses challenges. The fact that data is non-rival in consumption or usage and non-material complicates simple policies to prevent data from being harvested by foreign firms. The problem is the existence of cross-border spillovers in data analysis (Bergemann et al., 2019; Rubinfeld & Gal, 2017). Data spillovers mean that because consumers tend to be roughly similar in their make-up and psychology across jurisdictions, data from consumers in one country may be helpful in another protected jurisdiction. This gives an advantage to digital platforms operating in several countries. Thus, for example, Chinese-based digital platform firms may use data harvested in Kenya to design products and sell data to businesses targeting South African consumers without access to data of South African origin. Moreover, because data about one consumer helps to understand another consumer, the social value of data will exceed the private value of data. Hence, the cost of acquiring private data will be much less than the value of the data to the platform (Bergemann et al., 2019).

While this complexity of regulating data remains an obstacle in late industrialisation and an open challenge for digital industrial policy, there is, however, also an upside, namely that there is, at the time of writing, still policy space for novel digital industrial policies relating to data regulation and governance. As pointed out by Foster and Azmeh (2020, p. 1257), “*binding rules are relatively limited at the moment, there are significant grey areas in current rules, and slow progress at present within digital trade at the WTO and within RTAs*”. This creates scope—and urgency—for digital industrial policy in late-industrialising countries.

### Create a supportive environment for homegrown digital platforms

The third requirement for digitally relevant industrial policies in late industrialising countries is to help create a supportive environment for the emergence and growth of home-grown digital platforms—and of course, regulate these appropriately. This will require a focus on digital entrepreneurship, skills, infrastructure, and finance to develop digital entrepreneurial ecosystems. The purpose of such digital entrepreneurial ecosystems should be to nurture the growth of new ventures based on business models wherein data and consumer orientation are more centrally embedded than in traditional models. Such policies will require more research on the current state, drivers, and obstacles of the emerging digital platform landscape in developing regions. It should, of course, also be done to support—and not undermine—a country’s export superstars (see Freund & Pierola, 2015).

The literature on industrialisation in developing countries has focused mainly on the nature and potential of the new digital technologies and the requirements for late industrialising countries to benefit from these. It has neglected digital platforms. Nevertheless, this literature makes practical industrial policy recommendations relevant to creating a supportive environment for home-grown digital platforms. These include policies to promote (so-called twenty-first century) skills, digital entrepreneurship, complementary infrastructure, and global value chain integration (Matthess & Kunkel, 2020; OECD, 2017; UNCTAD, 2019).

As far as skills are concerned, Andreoni et al. (2021) stress that digital technologies are characterised by the “merging and overlapping of technologies” (“technology fusion”), which requires a premium on “foundational capabilities” to be able to absorb and implement new technologies. They argue against industrial policies that try to achieve some technology leapfrogging or policies that try to bypass manufacturing by trying to promote high-productivity services sectors (e.g., business, financial and transport services, and tourism) because these policies would require first that foundational capabilities are present. Lundvall and Rikap (2022: 11) consider such foundational capabilities—and their lack in late industrialising regions such as Africa as a significant obstacle and argues that “catching up requires a state with the capacity to build a strong domestic knowledge base, strong enough to negotiate the openness of the national – or supra-national – innovation system.” They are somewhat pessimistic that these countries will be able to achieve this, given the strong dominant position of global digital platforms.

As far as supporting infrastructure is concerned, there is a general and established recognition that the digital divide—reflected, for example, in low internet penetration rates and lack of significant broadband access—is one of the fundamental bottlenecks in the digitalisation of Africa’s industry. Cariolle (2021, p. 14) identifies the most severe shortcomings in African countries’ ICT / digital infrastructure to be high costs for internet usage,^17^ underdeveloped backbone backhaul and last-mile mobile networks, internet exchange points and data centres,^18^ lagging in SMC rollout, and lack of affordable and stable electricity. In Africa, however it is not only ICT (digital) infrastructure support that presents bottlenecks to digital platform participation but also physical infrastructures such as roads, rails, ports, and supportive logistical services, which has traditionally been an obstacle to GVC participation. Transport and logistics costs in Africa are three to four times higher than in the rest of the world (Plane, 2021). This reflects, in part, the continent’s adverse geography, which places it at large physical distances from global markets (Bosker & Garretsen, 2012).

When one considers the digital platform landscape, then mere internet access is not enough anymore: what matters for industrial competitiveness is bandwidth. The digital bandwidth gap between high-income countries and the rest of the world, measured in terms of the difference in average kilobits per second (kbps) bandwidth installed was 45 kbps in 2000, has grown to over 15,000 kbps by 2014 (Hilbert, 2016). This reflects that bandwidth expansion has underpinned the growth of digital platform firms in advanced economies and that a new digital divide is opening up between advanced economies and late industrialising countries.

While the USA and China dominate the global digital platform economy, there has been growth in Africa in the number of local (homegrown) digital platform firms, despite the skill and infrastructural bottlenecks mentioned in the previous paragraphs. Johnson et al. (2020) document the number and growth of digital platforms in several African countries in 2019, and Daramola and Etim (2022) studied the affordances that digital platforms offer users in Africa. Johnson et al. (2020) identified at least 365 digital platforms across eight countries with an average of 92,000 users per month and found that across Africa, the average user base is growing by 18% per annum. Around 82% of platforms are homegrown (i.e., originating in the country), and 20% are foreign—although foreign platforms were found to be capturing an increasing size of the market—as one would expect given the discussion in Sect. 3. Furthermore, Daramola and Etim (2022) reported that African digital platforms tend to lack affordances for multimodal interfacing, native language content and for enabling public–private partnerships.

Johnson et al. (2020) also found that homegrown platforms tend to focus mainly on the local market—few are expanding into other African countries, and the number of platforms is growing faster than the user base, which the authors see as a reflection of the fragmented nature of homegrown digital platforms.

The need for industrial policies to create a supportive environment for the emergence and growth of homegrown digital platforms in Africa is thus clear. Whether over time Africa can emulate China’s (as a developing country) success in the digital platform economy is, however, a cause for concern. Rikap and Lundvall (2020: 20) make the convincing conclusion that

“*The fact that China has been the only country that has successfully built competitors to GAFAM indicates that fostering firms that could challenge them would require a combination of long-term planning, entrepreneurial state intervention and big markets, particularly relevant when it comes to harvesting data […] In the case of Europe, Latin America and Africa, it would require a closer integration in terms of transnational and technological cooperation than what national governments have been ready to accept so far […] A huge coordination effort between third countries would be required.*”

## Conclusion

Digital platform capitalism and its consequences for already stagnating GVCs will make late industrialisation even more complicated than it ever had been. It has led to diminished expectations that many late industrialising countries had of the digital revolution (4IR). Consequently, industrial policy is becoming a battle for technological supremacy and control over fundamental digital assets—both tangible and intangible. The nature of GVCs is being altered by the rising dominance of digital platforms, and this complicates industrialisation by essentially requiring new paradigms for doing business and creating value. Moreover, regulations, standards, intellectual property, legislative measures, and international coordination are becoming more than ever crucial industrial policy tools.

In this, the policy and political processes of late industrialisers cannot remain behind. According to Kenney and Zysman (2016, p. 69), digital platform capitalism can lead to social and political upheaval, as was the case during earlier forms of capitalism. As they put it, “*The reality is that the winners and losers in markets depend on who can participate and on what terms. There are no markets, and no market platforms, without rules, but what happens to politics if important market rules are made unchallenged by the platform owners? Many political struggles will be waged over these rules, and those fights will define the market and society in a platform era*.” Late industrialising countries need digital industrial to ensure they get a say in establishing and policing the market rules for platform capitalism.

## Data Availability

There is no dataset associated with this paper.

## Footnotes

1 The primary mechanism has been through reducing frictions in international trade (Johnson & Noguera, 2017) and improving coordination and reducing location specificity of human capital (Autio et al., 2021). Lendle et al. (2016), for instance, show that the effect of distance is 65% smaller on a digital platform such as eBay.

2 The concept of a GVC is sometimes seen as too narrow, a criticism that has seen the concept of a Global Production Network (GPN) being elaborated. The latter focus on the broad networks involved in global production—see the discussion in Coe et al. (2008). It may ultimately be the more appropriate concept to evaluate the impact of digital platforms on trade. For a review of the GCV literature, see Kano et al. (2020).

3 The GVC participation rate can be measured from either the trade (Borin & Mancini, 2019) or the production side (Wang et al., 2017). Globally, the GVC participation rate has increased rapidly since the 1970s until the 2009/2010 global financial crisis, after which it stagnated. In 2020, the average global GVC participation, according to the trade measure, was 44.4% (ABD et al., 2021), up from 35% in 1970 (World Bank, 2020).

4 According to World Bank (2020) estimates, an 1% increase in GVC participation may raise a country’s per capita income by more than 1%. However, it also increases vulnerability, as GVCs were an essential channel of economic contagion during the COVID-19 pandemic (Coveri et al., 2020).

5 Digitalisation refers to “the proliferation and application of digital technologies in the economy” (Matthess & Kunkel, 2020, p. 2). Digital transformation refers to adapting business models through digitalisation and can be defined as “using digital technologies to develop new business models” (Broekhuizen et al., 2021, p. 847).

6 Platform markets and the network economies that drive their growth are not new. Before digital platforms, the most salient platform markets included newspapers, credit cards, barcodes, container shipping, real estate brokerages, shopping malls and stock exchanges (Eisenmann et al., 2011).

7 For example, Apple charges the developers of apps on its platform a 30% commission on their sales for using Apple’s consumer data. This resulted in around US $42.8 billion in revenue for Apple between 2007 and 2017 (Li et al., 2019).

8 The digital revolution, or the “second machine age”, refers to the rise in the importance of data, data-based technologies, and digital business models (Brynjolfsson & McAfee, 2016).

9 The Fourth Industrial Revolution (4IR) is often used interchangeably with “Industry 4.0”. It is, however, distinct, although related. “*The term Industry 4.0, originating from an initiative of the German government in 2011, refers to the intelligent networking of machines and industrial processes with the help of digital technologies and heralds the fourth industrial revolution*” (Das and Dey, 2021: 1293).

10 Cloud computing is where “computing is done on a network of off-site computing resources accessed through the Internet” (Byrne et al., 2018, p. 1).

11 Gartner IT’s Glossary defines big data as “high-volume, high-velocity and high-variety information assets that demand cost-effective, innovative forms of information processing for enhanced insight and decision making” (Gandomi & Haider, 2015, p. 138).

12 Computer Numerical Control (CNC) machining is the use of pre-programmed computer software to dictate “the movement of factory tools and machinery [...] to run them via software in repetitive, predictable cycles, all with little involvement from human operators” (Hess, 2017).

13 The energy needs of 3D printing are “100 times higher than traditional manufacturing” (Chan et al., 2018, p. 156).

14 Worldwide, over 2 million small businesses operated as 3rd party sellers on Amazon in 2020, the most -around 300,000—from the USA (in 2018) (Danziger, 2018). Sellers from 188 countries can, at the time of writing, sell directly on Amazon Marketplace, including sellers from 42 Sub-Saharan African countries.

15 Gatekeeping platforms are defined by the EC as those with “more than 45 million active monthly end users or more than 10,000 active yearly business users” (Cabral et al., 2021, p. 9).

16 Chinese firms are also not exempt from EU scrutiny and legislative measures, for instance, the case of Huawei and the provision of 5G telephone networks in Europe.

17 The cost of internet use in SSA is high due to monopolistic and oligopolistic market conditions and relatively high tax rates being levied on telecommunications operators (Cariolle, 2021).

18 Most African websites are hosted on European and American data centres (Cariolle, 2021).
